# Design and Development of a Topical Nanogel Formulation Comprising of a Unani Medicinal Agent for the Management of Pain

**DOI:** 10.3390/gels9100794

**Published:** 2023-10-02

**Authors:** Amit Sah, Geeta Aggarwal, Gaurav K. Jain, Syed Mohammad Abbas Zaidi, Punnoth Poonkuzhi Naseef, Mohamed S. Kuruniyan, Foziyah Zakir

**Affiliations:** 1 Department of Pharmaceutics, Delhi Institute of Pharmaceutical Sciences and Research, Delhi Pharmaceutical Sciences and Research University, Sector-3, M.B. Road, Pushp Vihar, New Delhi 110017, India; 2 Department of Pharmaceutics, School of Pharmaceutical Sciences, Delhi Pharmaceutical Sciences and Research University, Sector-3, M.B. Road, Pushp Vihar, New Delhi 110017, India; geetaaggarwal17@gmail.com; 3 Department of Moalajat (Internal Medicine), Hakim Syed Zia ul Hasan (HSZH) Govt. Unani Medical College, Bhopal 462003, India; 4 Department of Pharmaceutics, Moulana College of Pharmacy, Perinthalmanna Kerala 679321, India; drnaseefpp@gmail.com; 5 Department of Dental Technology, College of Applied Medical Sciences, King Khalid University, Abha 61421, Saudi Arabia; mkurunian@kku.edu.sa; 6 Department of B.Pharm (Ayurveda), School of Pharmaceutical Sciences, Delhi Pharmaceutical Sciences and Research University, Sector-3, M.B. Road, Pushp Vihar, New Delhi 110017, India

**Keywords:** Baboona, chamomile, *Matricaria chamomilla*, migraine, nanogel, nanoemulsion, pain, topical

## Abstract

The oil of the Unani medicinal herb Baboona (*Matricaria chamomilla*) has shown potential in the management of pain. However, predicaments such as poor skin penetration, skin sensitization, liable to degradation, and volatile nature restrict its use. Therefore, our group for the first time has developed a carrier-based delivery system to facilitate the direct application of chamomile oil to the forehead. The developed nanogel was characterized for physical parameters such as compatibility, TEM, and stability studies. Further, it was also evaluated for pH, viscosity, spread ability, and extrudability, as well as through texture analyses, in vitro studies, and skin irritation tests. The formulation was successfully developed with all the necessary attributes. The in vitro studies revealed the enhanced skin penetration of chamomile oil nanogel. The in vivo studies were also performed in chemically induced pain models, mimicking migraine. The studies show significant improvement of the pain threshold for chamomile nanogel when compared to the positive control group and the results were comparable to marketed diclofenac formulations. Finally, the encapsulation into nanogel reduced the skin irritation property. The nanogel formulation showed promising effects in the pain management of migraine.

## 1. Introduction

Migraine is a common neural condition characterized by chronic, pulsating pain, mostly experienced on one side of the head, i.e., unilateral attack [1]. The severity of headaches ranges from moderate to severe, usually persists for 4–24 h, and is associated with symptoms such as nausea, vomiting, photophobia, or phonophobia. It is most common in children and younger individuals, especially in women [2].

According to the World Health Organization, migraine affects approximately 12% of the global population, 17% of women, and 6% of men [3]. It severely affects quality of life and puts a burden on individuals due to the high treatment costs. Although many treatments are available for pain management, they exhibit limited efficacy due to significant side effects, toxicity, and unfavorable physicochemical characteristics [4]. In the search for alternate therapies, herbal plants and oils have been explored as they contain comparatively far safer compounds. The popularity of natural medicine is continuously increasing, and it has emerged as a globally sought-after approach. Unani is an ancient medicinal system of medicine that is widely practiced in India as well as in certain other countries. The application of herbal drugs into nanotechnology is a novel approach to magnify the solubility, absorption rate, and permeation of herbal medicine, which demonstrates the high bioavailability and therapeutic potential of these medicinal plants [5].

Baboona (*Matricaria chamomilla*) is a very promising Unani medicinal herb used orally as well as locally to treat many disorders including various kinds of headaches, particularly migraine [6]. Research regarding its formulation and application in medicine is rare, and it is used for its soothing qualities as a sedative, mild analgesic, and sleep medication. The main constituents in chamomile essential oil (CO) are chamazulene, apigenin, and bisabolol which possess properties of pain management. There are many reports which claim that CO possesses properties for the treatment of migraine [7]. The apigenin and its derivatives act as a COX-2 inhibitor and possess anti-inflammatory effects due to the inhibition of endogenous prostaglandin E2 (PGE2) levels in RAW 264.7 macrophages [8]. It also acts as a neuroprotective agent because of the inhibition of nitric oxide synthase and subsequently leads to inhibited nitric oxide levels, which play an important role in inducing acute to chronic migraine attack [9].

However, pure essential oil has irritation potential and cannot be applied directly to the forehead. Additionally, CO is volatile, with poor tissue permeability and high degradation. Therefore, its beneficial therapeutic properties require a delivery approach that is able to improve its penetrative power, negate its volatility, and enhance the therapeutic effectiveness of CO [6]. This could be achieved by delivering CO in a sustained manner by using a delivery carrier such as nanoemulsion-based gel. Nanoemulsion formulations possess unique properties such as high biocompatibility, ease of surface modification, and smaller size [10]. Nanoemulsion-based formulations are effective fortreating migraine, as they enhance the therapeutic efficacy and tolerability of antimigraine drugs [11]. Moreover, a nanoemulsion-based nanogel offers high patient acceptability, as it is non-invasive and easily applicable [12]. Despite the numerous therapeutic benefits of CO in migraine, nobody has ever attempted to enhance its efficacy using a suitable carrier system.

In our work, we propose the formulation of a CO-loaded nanoemulsion (CON) and its conversion into gel (COG), which is expected to reduce skin irritation and enhance the therapeutic activity of CO. The study will be supplemented with characterization studies to confirm the suitability of the formulation, as well as in vitro release and permeation studies and in vivo pharmacodynamic studies in order to demonstrate performance attributes, followed by in vitro skin irritation and stability studies, which will address the physical stability and acceptability of the formulation.

## 2. Results and Discussion

### 2.1. Construction of Pseudo-Ternary Phase Diagrams

**Figure 1:**
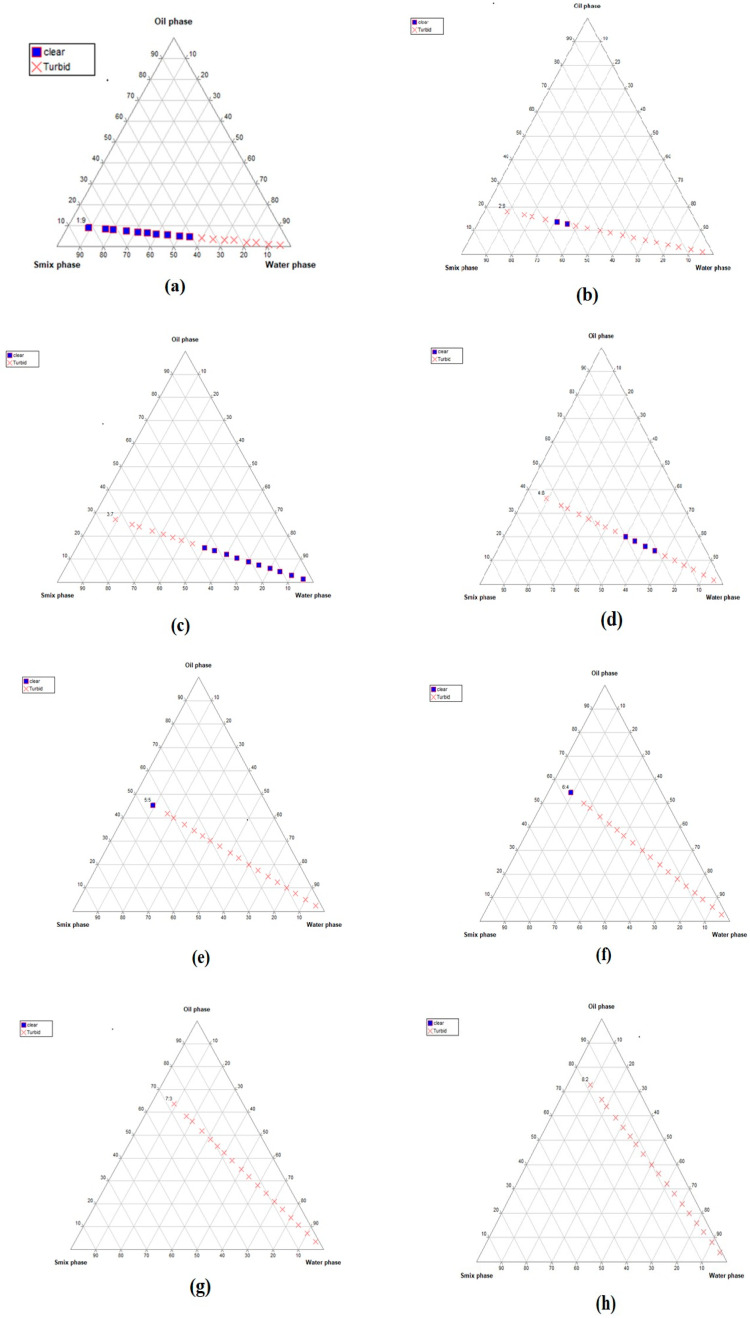

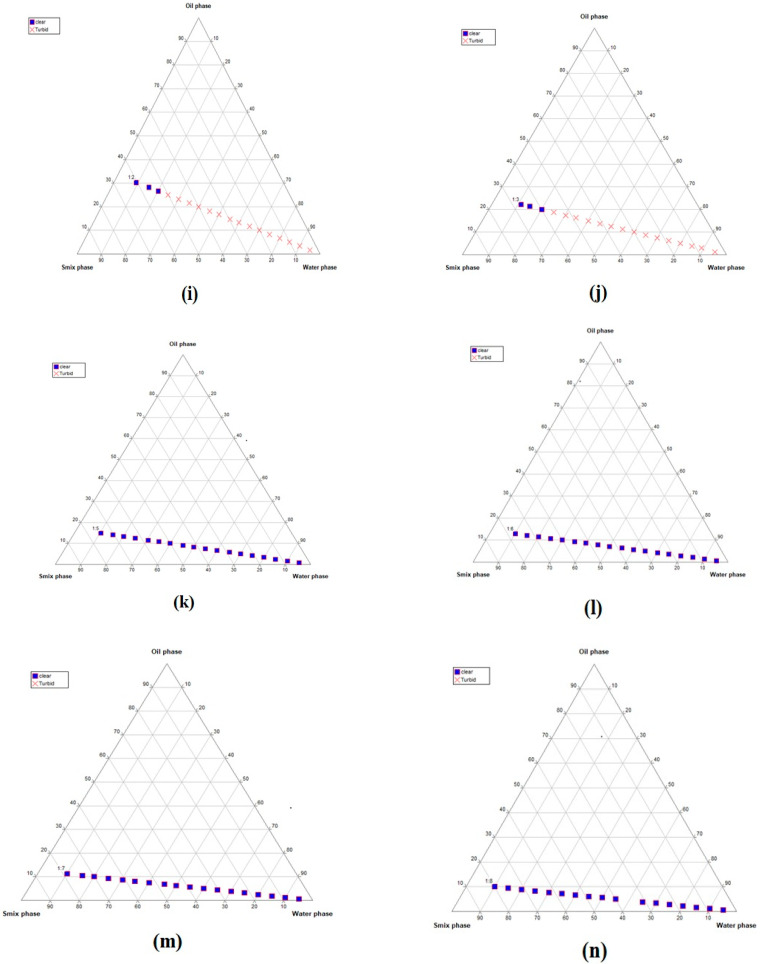
Pseudo-ternary phase diagrams of the developed nanoemulsion formulations using different oil:Smix ratios: (**a**) 1:9, (**b**) 2:8, (**c**) 3:7, (**d**) 4:6, (**e**) 5:5, (**f**) 6:4, (**g**) 7:3, (**h**) 8:2, (**i**) 1:2, (**j**) 1:3, (**k**) 1:5, (**l**) 1:6, (**m**) 1:7, and (**n**) 1:8. The figure shows oil, surfactant:cosurfatant, and water in each corner with 100% of each component. The blue dots indicate clear dispersion, whereas red crosses denote turbidity. It can be seen that the nanoemulsion forming zone (represented by blue dots) is larger in images (**k**–**n**), suggesting stable formulation.

All the nanoemulsions were prepared usingthe aqueous titration method, as shown in Figure 1. The nanoemulsion forming zone was marked on pseudo-ternary phase diagrams. The pseudo-ternary phase diagrams with the largest nanoemulsion-forming zones were selected for the preparation of nanoemulsions. It was observed that the Smix, comprising PEG 400/Tween 20 at a ratio of 1:1, revealed the formation of a nanoemulsion. Further, the most prominent nanoemulsion zone was seen in the oil:Smix ratio of 1:5.

### 2.2. Thermodynamic Stability Studies of Developed Nanoemulsion

The stability studies of different chamomile-loaded nanoemulsion formulations are shown in Table 1. The CONs that were physically unstable showed signs of phase separation and were therefore rejected.

### 2.3. Characterization of Selected Nanoemulsion Formulations

#### 2.3.1. Particle Size, Zeta Potential, and Polydispersity Index (PDI)

**Figure 2:**
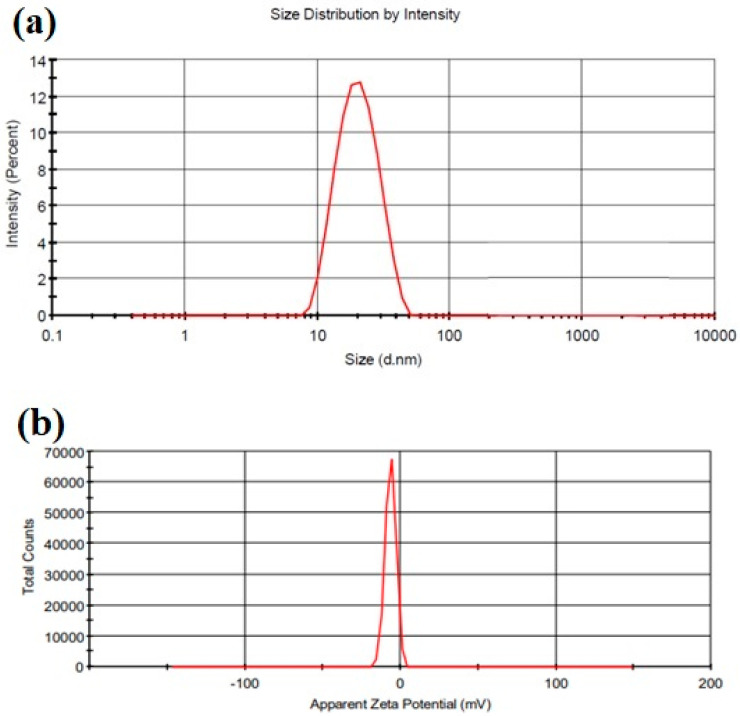
(**a**) Droplet size distribution by intensity; (**b**) zeta potential graph.

The particle size, zeta potential, and polydispersity index of the optimized CONs are 22.95 nm, −6.27 mV, and 0.335, respectively, as represented in Figure 2.

#### 2.3.2. Transmission Electron Microscopy

**Figure 3:**
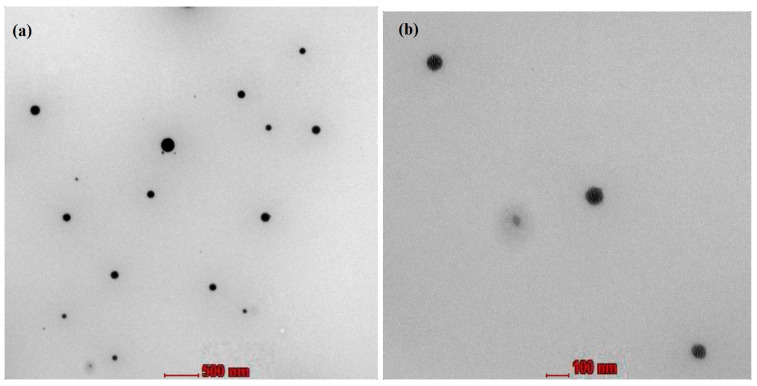
Transmission electron microscope images of nanoemulsion droplets at a magnification of (**a**) 11,000× (scale in the image represents 500 nm) and (**b**) 45,000× (scale in the image represents 500 nm).

Transmission electron microscopy (TEM) was performed in order to study the morphology and structure of the optimized CONs. The formulations were observed at magnification 45,000× and 11,000× under TEM. From the TEM images, we can conclude that globules were well-formed and spherical. No aggregation was observed. The globules observed in the TEM images were found to be less than 100 nm (Figure 3).

#### 2.3.3. Compatibility Studies

**Figure 4:**
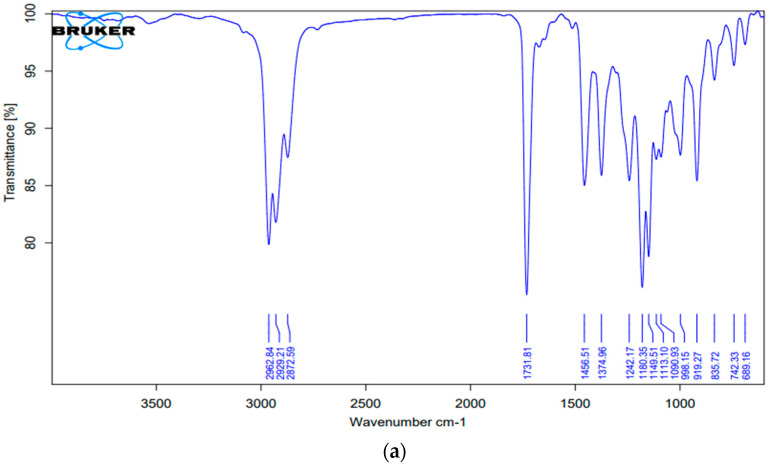

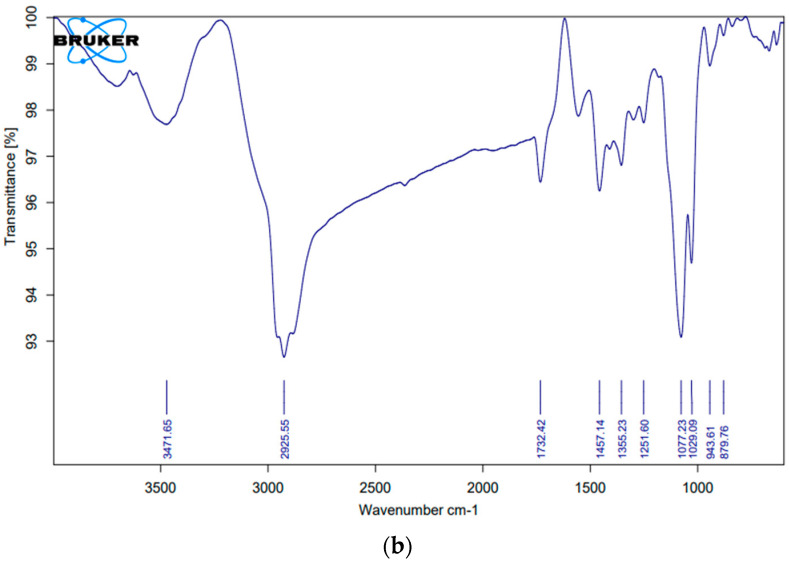
Fourier transform infrared spectra of (**a**) pure chamomile oil and (**b**) chamomile oil-loaded nanoemulsion.

Structural compatibility was further observed for CON and CO with the help of the FT-IR spectra (Table 2). The FT-IR spectrum of pure CO was compared with that of CON. Since there was no major shift in the peaks of CO-loaded nanoemulsion, this indicates no chemical incompatibility or interactions in the drug-excipient combination, as shown in Figure 4a,b.

### 2.4. Preparation and Evaluation of Nanogel

The prepared nanogel was evaluated for various parameters to uncover whether the final optimized COG was suitable for topical application.

#### 2.4.1. pH Measurement

The pH value of the nanogel was found to be 5.8 ± 0.03, as determined by the pH meter.

#### 2.4.2. Viscosity

**Figure 5:**
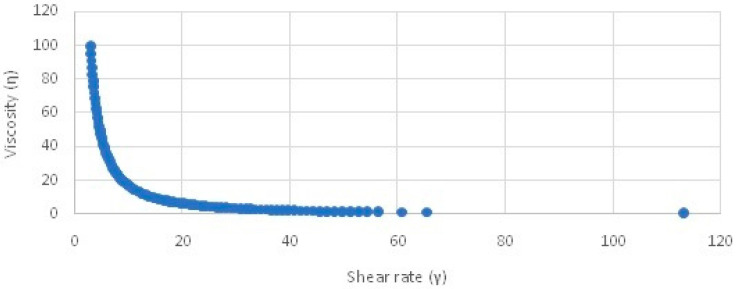
Viscosity vs. shear rate graph of the developed chamomile nanogel.

The values for viscosity shown in Figure 5 indicate a decrease in viscosity upon increasing the shear rate from 1 to 100 s^−1^. This indicates the shear-thinning or pseudoplastic behavior of the prepared formulations.

#### 2.4.3. Spreadability and Extrudability Tests

It was observed that the gel formulation showed good spreadability (21.66 g∙cm/s), which is required for easier topical application. Similarly, the extrudability of the formulation was found to be 89.9%. Sufficient extrudability is an important requirement for the easy removal of the gel from the tube while it is being used by the patient.

#### 2.4.4. Texture Analysis

**Figure 6:**
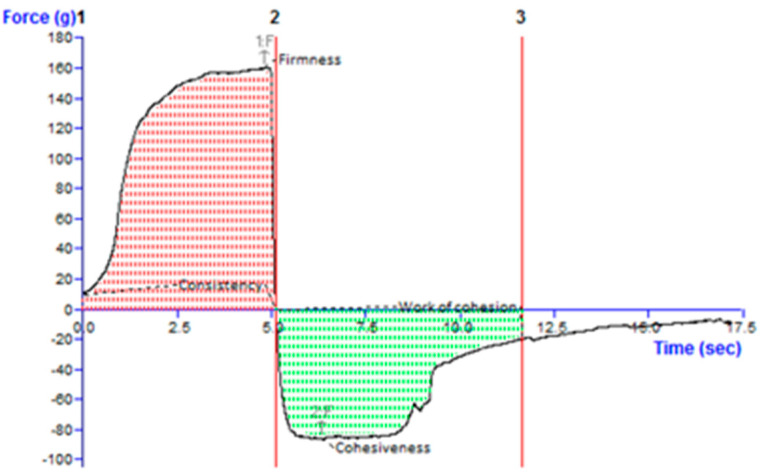
Texture profile analysis graph of the developed chamomile oil nanogel. Line 2 indicates the firmness value when pointing up, and when pointing down, it indicates cohesiveness. The area between 1 and 2 is consistency and between 2 and 3 is cohesiveness.

The firmness, consistency, cohesiveness, and cohesion function were assessed as texture parameters of the formulations, as shown in Figure 6, and were found to be 161.14 g, 618.77 g∙s, −86.20 g, and −392.94 g∙s, respectively.

### 2.5. Analytical Method Development

The calibration curve was constructed for apigenin, the major chemical component present in CO. The linear regression analysis data showed a good linear relationship (r^2^ = 0.9979) concerning the peak area in the concentration range of 10–50 ng/μL per spot. The values of slope and intercept are 0.0006 and 0.0001, respectively.

### 2.6. In Vitro Drug Diffusion Study

**Figure 7:**
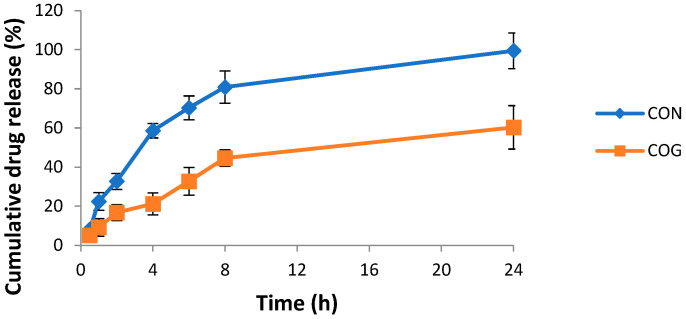
Cumulative percent drug release vs. time graph of the chamomile oil nanogel (COG) vs. chamomile oil nanoemulsion (CON) in a phosphate buffer of pH 6.

An in vitro diffusion study was carried out in a dialysis membrane bag for 24 h and showed maximum drug release (99.5 ± 9.1%) for CONs, as shown in Figure 7. When the formulation was loaded into the gel, then the release was sustained with only a 60 ± 11.3% release over 24 h.

### 2.7. Drug-Release Kinetics

In vitro study data were fitted in different models, such as zero-order and first-order, as well as into a Higuchi plot, Korsmeyer–Peppas plot, and Hixson–Crowell plot. The Korsmeyer–Peppas plot out of all the above showed the highest linearity, which was interpreted via regression coefficients, as shown in Table 3. It was observed from the analysis that an R^2^ value of 0.9051 was obtained in the Korsmeyer–Peppas plot with an n value of 0.323, which reveals the Fickian diffusion as shown.

### 2.8. Skin Penetration Study

**Figure 8:**
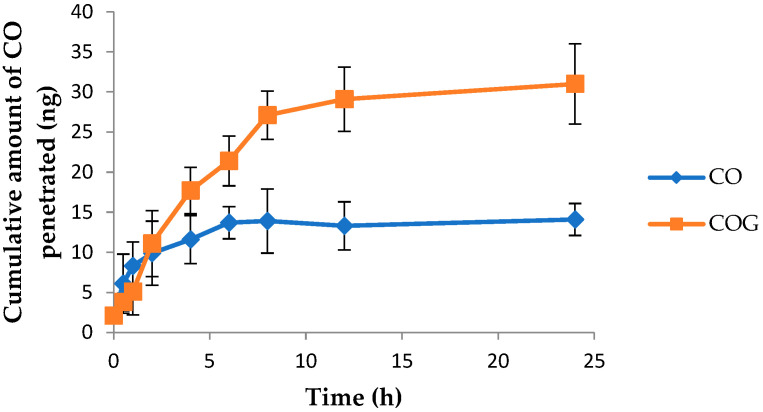
Graph depicting the cumulative amount of pure chamomile oil (CO) and chamomile oil from nanogel (COG) penetrating excised rat skin vs. time.

The skin penetration ability of the CO in the formulation was also assessed. It was observed that only about 14 ± 2.1 ng of CO was penetrated when used alone(Figure 8). On the other hand, this number rose considerably when incorporated into a nanogel formulation, with about 70% of the CO penetrating the skin within 24 h. This shows the success of the formulation.

### 2.9. In Vivo Studies

#### 2.9.1. Tail Flick Test

The animals in the control group were subjected to a heat source and the time for latency was noted throughout the test period. No significant difference in reaction time was noted. The second group of animals was treated with diclofenac sodium and the latency time was increased considerably (7.21 ± 0.97), which was suggestive of an increased pain threshold. Furthermore, the group treated with a topical chamomile formulation was also tested and showed a significant increase (6.88 ± 0.81, *p* < 0.001) in tail flick latency, somewhat similar to the response given by the standard group (Table 4). However, the insignificant difference between standard treatment and chamomile formulation as depicted by the percent inhibition in analgesia suggests comparable analgesic activity.

#### 2.9.2. Acetic Acid-Induced Writhing Test

The administration of acetic acid in Wistar rats induces pain, which had been correlated with pain suffered in migraine. The irritation generated viaacetic acid in the abdominal region of rats causes it to constrict their abdomen or stretch their hind limbs, which is known as writhing. It was observed that there was a significant writhing count after the rats were exposed to acetic acid during the experiment, confirming the successful induction of migraine. However, upon treatment with standard and test formulations, the exposed rats showed a significant decrease (*p* < 0.001) in writhing count compared to the acetic acid-exposed group (control), as shown in Table 5. We can conclude that the application of chamomile oil nanogel resulted in significantly (*p* < 0.001) reduced writhing, as compared to the control group. The response shown by the test group was almost similar to the responses exhibited through application of the standard treatment, diclofenac sodium, as suggested by their protection percentages.

#### 2.9.3. Light/Dark Box Model

**Figure 9:**
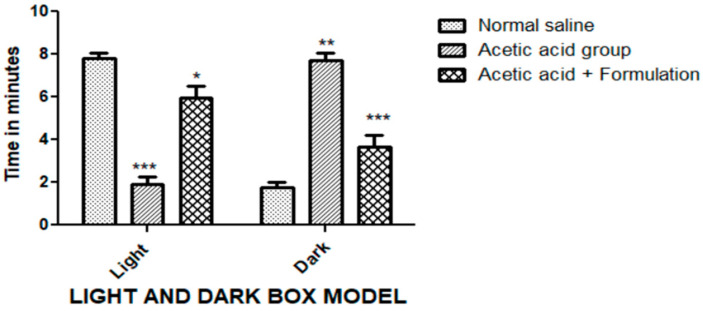
Anxiolytic effect via the light and dark box model. The duration of stay ofthe rats in both light and dark compartments was tested for different treatment groups. The data represented are mean ± SEM. Where *** *p* < 0.001, ** *p* < 0.01, and * *p* < 0.05. A two-way ANOVA with Tukey’s post hoc test was used for statistical analysis.

Nitroglycerine exposure to experimental animals showed a marked rise in the time spent in the dark box (420 ± 18.27; *p* < 0.01) and a decline in time spent in the light box (114 ± 18.27; *p* < 0.001), compared to negative control rats; stating that nitroglycerine successfully induced ananxiolytic effect (Figure 9). However, the administration of the COG formulation showed a significant decline in the time spent in the dark box (228 ± 28.65; *p* < 0.001), as well as a rise in the time spent in the light box (360 ± 28.65; *p* < 0.05) upon comparison with the positive control group. The number of transitions during the experiment was the highest in the control group and showed a significant decline in thepositive control group, suggestive of depressive behavior. However, the topical COG showed a significant elevation in the transitions.

### 2.10. Skin Irritation Test

**Figure 10:**
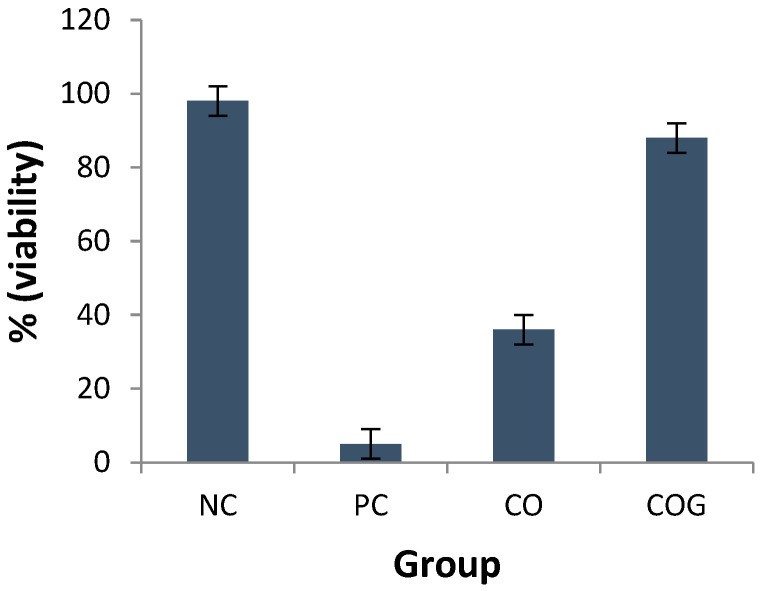
The in vitro skin irritation test expressed as percentage viability for different treatment groups; NC, negative control; PC, positive control; CO, pure chamomile oil; COG, chamomile oil nanogel.

From the graph (Figure 10), it is clear that maximum viability is observed for negative control suggestive of negligible skin irritation. On the other hand, positive control showed a viability of only 5%, which suggests toxicity to human epidermal cells. CO exhibited a reduced viability score of 36%, which depicts its irritant behavior. The loading of CO into gel significantly reduced its irritancy, which suggests that it is safe for topical application.

### 2.11. Stability Study

The appearance, phase separation, pH, and percent transmittance of formulated nanogel were observed to be consistent with no signs of separation and deterioration over a period of 60 days (Table 6). The results indicated that the optimized formulation was found to be physically and chemically stable during the study period.

### 2.12. Discussion

For the development of nanoemulsion, PEG 400 and Tween 20 were selected as the surfactants and co-surfactants, respectively, as they exhibited good solubility with CO. Different Smix ratios and oil:Smix ratios were used for the development of pseudo-ternary phase diagrams. Only four oil:Smix ratios with an Smix component of 1:1 showed nanoemulsion formation, namely, 1:5, 1:6, 1:7, and 1:8. The phase diagrams were compared in order to identify a maximum nanoemulsion region to select the optimized formulation, which was found to be the 1:5 oil to Smix ratio. The selected formulations were further screened using thermodynamic stability testing to choose the most stable formulation and eliminate metastable formulations. The optimized formulation (1:5) was further subjected to the characterization of various parameters. The particle size, PdI, and zeta potential were found to be satisfactory. The nanoemulsion of droplet size 22 nm was further confirmed with TEM studies. A chamomile oil-excipient compatibility study is a crucial step to decide the long term stability of the formulation. From the FTIR spectra, it is evident that the characteristic absorption peaks of chamomile oil were also observed in nanoemulsion, such as 2925.55 cm^−1^ (-CH2- aliphatic asymmetric), 1732.42 cm^−1^ (C=O), 1457.14 cm^−1^ (=C-H scissor), and 1355.23 cm^−1^ (C-O) [13], which strongly suggest that the chemical structure of chamomile oil is not altered and thus reveals the absence of any interaction.

The next step in formulation development was the preparation of nanoemulsion-based gel, i.e., nanogel. Carbopol 940 was selected as the gelling agent for the preparation of gel because of its excellent gel-forming property at low concentrations. Additionally, it is biodegradable, biocompatible, and non-toxic to the human body [14]. Nanoemulsions as such are not suitable for topical application because of their higher flowability and poor viscosity, which may also affect the contact time of the formulation with the skin. The conversion of a nanoemulsion into gel would improve the viscosity, consistency, and applicability of the formulation at the site of application. This would also improve the contact time and the overall penetration of the nanoemulsion formulation into the skin and ultimately improve the efficacy of the formulation. A 0.5% *w*/*w* Carbopol gel was incorporated into the nanoemulsion at a ratio of 1:1. The viscosity analysis of the developed nanogel has shown shear thinning behavior, which is favorable. A viscous gel-like consistency on rubbing action will reduce its viscosity, increase spreadability, and thus enhanced penetration can be expected which is proven by skin penetration studies. The nanosize of the carrier system coupled with the skin penetration properties of nanoemulsion components and Carbopol gel have facilitated enhanced penetration through the skin barrier. Moreover, the sustained release of CO from the nanoemulsion gel will enable the longer residence of CO in the skin.

Furthermore, the pharmacological activity of the developed formulation was tested against migraine. The tail flick method was used as an indication of centrally acting analgesic activity [15]. Since migraine is associated with throbbing pain, a thermally induced hyperalgesia method was used to monitor the protective effects of the chamomile formulation. It can be seen that, in comparison to the control, a significant delay in the reaction time of with drawing the tail was noted for both standard and test formulations. This is suggestive of the anti-nociceptive property of chamomile.

Acetic acid is known to induce pain via the release of chemicals that trigger nociceptors, characterized by episodes of retraction of the abdomen and the stretching of hind limbs. The signal is transmitted to the central nervous system, which further causes the release of prostaglandin and contributes to the increased sensitivity to nociceptors [16]. This model is used for detecting the peripheral analgesic activity. A significantly reduced number of writhings observed after the application of the chamomile formulation is suggestive of analgesic activity. It is known that migraine patients are often hypersensitive to light; therefore, a light–dark box model was a requisite model for studying the anti-migraine properties of the developed formulation. Nitroglycerine, a known nitric oxide donor, evokes the trigeminovascular system and develops hyperalgesia and photophobia, which are characteristic of migraine [17]. During the study, it was found that pre-treatment with chamomile formulation reduced photophobia as compared to other groups, proving that chamomile suppresses nitric oxide, which is responsible for the inflammation and induction of migraine. Our results confirm previous findings that chamomile possesses antimigraine potential through the inhibition of nitric oxide release [7,18].

Further, chamomile also increased the tolerance to pain via both central and peripheral mechanisms. Since migraine is associated with both peripheral and centrally acting sensitizations [19], topical chamomile nanogel formulation can be effectively used for the treatment of migraine. However, a major drawback of chamomile oil is skin sensitization potential. Therefore, the oil was encapsulated in a nanogel formulation. Skin irritation potential was tested using a reconstituted human epidermal skin model, as per the OECD TG 439 protocol. The principle for this test is an MTT assay, where viable cells convert MTT into blue formazan salt [20]. Therefore, the higher the color intensity at 570 nm, the higher the viability of cells. The results of the test conclude that the formulation is safe for topical use.

## 3. Conclusions

The chamomile oil-loaded nanogel formulation was successfully developed with a uniform droplet size of less than 100 nm. The release studies show that the incorporation of chamomile oil intonanoemulsion has improved aqueous solubilization and restricted release when converted into a gel. This has provided a sustained release of chamomile oil. Increased skin penetration was observed due to the nanosize of the droplets coupled with the presence of skin permeation ingredients in the formulation. The shear thinning properties of the gel facilitated better dermal penetration due to the rubbing action, which reduced the viscosity. Finally, the in vivo studies revealed significant anti-nociceptive properties and an increased pain threshold for the developed formulation, suggesting analgesic activity. It was also observed that the formulation reduced photophobia in nitrolycerine-induced migraine models, which suggests thatprevents migraine by inhibiting the release of nitric oxide. However, further studies are still needed to confirm anti-migraine properties. Nonetheless, the studies conclude that the developed topical chamomile oil nanogel is safe for application to the forehead and can be used for analgesia in migraine.

## 4. Materials and Methods

### 4.1. Materials

Chamomile oil was procured from Vaadi Herbals, Pvt. Ltd. (Delhi, India) Apigenin, acetic acid, chloroform, toluene, and Tween 20 were procured from Sigma-Aldrich (St. Louis, MO, USA). Carbopol 940, Polyethylene glycol (PEG 400), triethanolamine, and methanol were procured from LobaChemie, Pvt. Ltd. (Mumbai, India). The dialysis membrane (size: 14,000 Da; diameter 17.5 mm) was procured from Hi-Media Laboratories, Pvt. Ltd. (Kennett Square, PA, USA).

### 4.2. Method of Preparation

#### 4.2.1. Selection of the Nanoemulsion-Forming Zone viaPseudo-Ternary Phase Diagrams

For the pseudo-ternary phase diagrams, surfactant/co-surfactant mixtures (Smix) of Tween 80/span 80, Cremophor^®^ RH40/span 80, and PEG 400/Tween 20 at different ratios (1:1, 1:2, 2:1, 1:3, and 3:1) were prepared. These pseudo-ternary phase diagrams were composed of fixed ratios of chamomile oil and Smix, namely, 1:9, 1:8, 1:7, 1:6, 1:5, 2:8 (1:4), 1:5, 1:3, 1:2, 3:7 (1:2.3), 4:6 (1:1.5), 5:5 (1:1), 6:4 (1:0.7), 7:3 (1:0.43), 8:2 (1:0.25), and 9:1 (1:0.1) with water. The mixtures of the Smix ratio were selected based on their combined HLB values. According to the method, CO and Smix were mixed together at room temperature to obtain the organic phase. Then, the water was added dropwise to the organic phase using a vortex shaker and was visualized against a light and dark background and observations were carried out for transparent formulation [21]. The results were plotted in pseudo-ternary phase diagrams using PCP-triangular software.

#### 4.2.2. Preparation of Nanoemulsion

Pseudo-ternary phase diagrams that resulted in a maximum nanoemulsion formation zone were selected for the preparation of nanoemulsions. The composition of nanoemulsions in terms of CO:Smix:water ratios were selected from the nanoemulsion forming zones obtained from the pseudo-ternary phase diagrams. All the CON formulations were prepared via the aqueous titration method [21].

#### 4.2.3. Thermodynamic Stability Studies of Developed Nanoemulsions

The thermodynamic stability testing of the developed CONs was carried out via aheating–cooling cycle, centrifugation, and a freeze–thaw cycle [21].

(1) Heating–cooling cycle: The samples were subjected to six storage cycles at alternate temperatures, i.e., refrigerator temperatures of 4 °C and at 45 °C, for 48 h each, and the formulated CONs were examined for stability (transparent with no phase separation).

(2) Centrifugation test: The formulated CON was centrifuged at 3500 rpm for 30 min and observed for transparency and the absence of phase separation.

(3) Freeze–thaw cycle: Three freeze–thaw cycles of the CONs between −21 °C and +25 °C for 48 h were performed and observed for transparency and the absence of phase separation.

#### 4.2.4. Characterization of Optimized Nanoemulsion Formulations

##### Particle Size, Zeta Potential, and Polydispersity Index

The particle size, zeta potential, and polydispersity index (PDI) of samples were evaluated by the zetasizer apparatus (Malvern zeta sizer; Nano-ZS90). The evaluation was carried out at 25 °C at an angle of 90°. The droplet size isshownas the z-average diameter (d.nm) and the particle size distribution wasevaluated via thepolydispersity index (PDI). Samples were analyzed in triplicate [22].

##### Transmission Electron Microscopy (TEM)

For morphological aspects, CON formulations were further studied using transmission electron microscopy (TEM). The samples were placed on a carbon-coated grid and then stained with 1% phosphotungstic acid, following which theywereleft at room temperature for drying. Images at different magnifications under TEM were observed [23].

##### Compatibility Studies

The compatibility between CO and other components of the nanoemulsion was studied viaFT-IR spectroscopy (Agilent Technologies Cary 630). The spectrum of CO and CON was recorded as being inthe region of 4000 to 400 cm^−1^ [24].

#### 4.2.5. Preparation of the Nanogel

A total of 0.5% *w*/*w* Carbopol 940 gel was prepared viasoaking in distilled water and was left overnight. Later, 1–3 drops of triethanolamine were added throughstirring, and then, the prepared nanoemulsion was incorporated ata ratio of 1:1. Methylparaben sodium (0.2% *w*/*w*) was also added via constant stirring [22].

#### 4.2.6. Characterization and Evaluation of Nanoemulsion-Based Chamomile Nanogel

##### pH Measurement

The pH of COG was determined using a calibrated digital pH meter.

##### Viscosity

The viscosity was measured by a Rheolab QC rheometer using Rheoplus/32, v 3.61 software. The viscosity of the developed COG formulations was measured using a shear rate ranging from 1 to 100 s^−1^ for 3 min using 5 g of gel at 25 ± 2 °C. The shear strain exerted by the formulations due to the application of shear stress can be calculated using the following equation [25].

Viscosity = Shear stress /Shear strain

##### Spreadability

A sample of 5 g of developed COG was pressed between two slides and left for 5 min. The diameters of spread circles were measured in cm and the spreadability was calculated using the following formula:

S = ML/T

where S indicates the spreadability (g∙cm/s), M indicates the mass (g), L indicates the length (cm), and T indicates the time (s) [26].

##### Extrudability

The developed COG formulations were placedinto clean collapsible tubes with 5 mm openings. These were then sealed with the help of heat. A constant load of 500 g was placed on the tube to release the gel from the tube. The amount of the extruded gel was collected, weighed, and the extrudability percentagewas calculated [26].

##### Texture Analysis

Texture profile analysis was carried out using a texture analyzer. The textural/mechanical properties of different COG formulations were measured using a TA.XT PLUS texture analyzer (Stable Microsystem, Surrey, UK) in texture profile analysis mode. This helps in the determination of textural parameters, such as firmness, consistency, and cohesiveness [27].

#### 4.2.7. Analytical Method Development

Pre-coated HPTLC plates (silica gel 60 F254) of size 20 × 20 cm were used for chromatographic development. The twin trough glass chamber (CAMAG) was pre-saturated with a mobile phase consisting of toluene, ethyl acetate, and formic acid at a ratio of 4.5:3.5:0.2 (*v*/*v*/*v*) for 20 min to ensure the uniform distribution of solvent vapors. Samples were applied using a CAMAG Linomat V applicator viaa microliter syringe, witha capacity of 100 µL as narrow bands of 6 mm in width. The chromatogram was developed to up to 80% of plate height through the linear ascending development technique at room temperature (25 ± 2 °C) and a relative humidity of 55 ± 5%. The plate was removed, air-dried, and then derivatized with anisaldehyde sulfuric acid spraying reagent. The prepared chromatogram was dried in an oven at 60 °C for 5 min. Densitometric analysis was performed at 570 nm with a Camag TLC scanner III. The slit dimensions were 5 mm × 0.45 mm and the scanning speed of 20 mm s^−1^. Different volumes of stock solution 2, 4, 6, 8, and 10 µL were spotted in triplicate on a TLC plate to obtain concentrations of 10, 20, 30, 40, and 50 ng per spot of apigenin. The data of peak areas and corresponding concentrations were treated via linear least-square regression analysis [28,29].

#### 4.2.8. In Vitro Studies

The in vitro release of the developed COG and CON was performed using a dialysis membrane. A beaker placed on a magnetic stirrer was filled with phosphate buffer of pH 6 (150 mL) and kept at 37 ± 1 °C. A 1 g gel sample was placed in a dialysis membrane bag. A total of 1 mL of each sample was removedat 0.5, 1, 2, 4, 6, 8, and 24 h and at the same time replaced with an equal volume of dissolution medium. All samples were analyzed for drug content by using HPTLC at a wavelength of 570 nm [30].

#### 4.2.9. Drug-Release Kinetics

The release kinetics of the developed COG was obtained from the in vitro drug-release data plotted in various kinetic models using the DD Solver add-in program. The drug-release kinetics were studied for various models and plots, such as zero-order, first-order, Higuchi, Korsmeyer–Peppas, and Hixson–Crowell [30].

#### 4.2.10. Skin Penetration Study

The skin penetration study was carried out as per a previously reported procedure with slight modifications [31]. The study was performed using a Franz diffusion cell with a diffusion area of 3.3 cm^2^ and a volume of 60 mL using the dorsal skin of Wistar rats. PBS of pH 7.4 was used as dissolution medium, at a temperature of 37 °C and a stirring speed of 300 rpm. The excised skin sample was sandwiched between the donor and receptor compartments. The formulation (1 g) was placed on the donor area and the samples were withdrawn at pre-determined time intervals and analyzed using HPTLC.

#### 4.2.11. Animal Studies

Adult albino Wistar rats of either sex weighing 200–250 g were selected for the study. The rats were acclimatized for a week before the initiation of the experiment. The rats were kept under laboratory conditions, i.e., temperatures of 25 ± 2 °C, relative humidity of 45 ± 5%, and a photoperiod of 12 h. For the topical administration of nanogel, the hair of the interscapular region of rats (3 cm^2^) was removed using hair removal cream. After the application of COG to the shaved region, the area was protected using a nylon mesh.

##### Tail Flick Test

The rats were divided into three groups, with six rats in each group, and administered the following respective treatments: the control group received only normal saline (1 mL/kg I.P.), the standard group received diclofenac gel (5%), and the test group received topical COG (100 mg/rat). The test was performed with a digital tail-flick instrument that allowed the automatic recording of the latency of the tail-flick response to radiant heat [32]. The number of seconds elapsing between the activation of the heat source and the rat flicking its tail away (latency) was recorded. To minimize tissue damage, a maximum latency of 15 s was imposed. The test was performed before and 30 min after treatment. The inhibition percentagein analgesia was calculated using the following formula:

$$\mathrm{Percentinhibition}=\frac{\mathrm{Posttreatmentlatency}-\mathrm{pretreatmentlatency}}{\mathrm{cutofftime}-\mathrm{pretreatmentlatency}}\times 100$$

##### Acetic Acid-Induced Writhing Test

The analgesic activity of formulation was evaluated using the acetic acid-induced writhing method [33]. For the study, twenty four Wistar rats were randomly divided into three groups and administered the respective treatments: the control group was administered normal saline (1 mL/kg I.P.), the standard group was administered diclofenac gel (5%), and the test group was administered topical COG (100 mg/rat). After 30 min, writhing was generated via acetic acid administration 0.3% (10 mL/kg I.P.). After 5 min, the number of writhings was counted for a total period of 10 min. The protection percentage was calculated using the formula:

$$\mathrm{Percentprotection}=\frac{\mathrm{Numberofwrithingsincontrol}-\mathrm{numberofwrithingsintest}}{\mathrm{numberofwrithingsincontrol}}\times 100$$

##### Light/Dark Box Model

There are two compartments in a rectangular box, light and dark. The light compartment was illuminated with a bulb and the top was left uncovered and the dark compartment was painted completely black and covered on all sides. A gate between the two chambers allowed the easy movement of rats between the two compartments. The rats were divided into three groups and the respective treatments were administered: the negative control group received only normal saline I.P., the positive control group received nitroglycerine i.v. (10 mg/kg), and the test grouprecieved topical COG (100 mg/rat). After 30 min, intravenous nitroglycerine (10 mg/kg) was administered to rats to induce migraine [34]. The animals were then placed in the middle of the two chambers and observed in order to measure the length of time they remained in the lit compartment. The time spent was calculated usingthe ratio of time spent by the rat in the lit compartment to the total period for which was observed.Furthermore, the number of transitions between the two compartments during the experiment was also recorded [35].

#### 4.2.12. Skin Irritation Test

The irritation potential of the COG topical formulation was evaluated by an in vitro skin irritation test. A previously reported method was followed [31], where artificial human epidermal cells were incubated as per the procedure. The test was performed in three groups comprising the negative control (using phosphate-buffered saline (PBS)); the positive control (using 5% SDS and CO) and the test group (using chamomile nanogel). The treatments were added to epidermal cells and incubated for 42 min at room temperature. They were then rinsed with PBS and transferred to a growth medium for incubation at 42 h, 37 °C, 5% CO_2_, and ≥95% humidity. The tissues were then placed in MTT solution for 3 h, 37 °C, rinsed with 300µL PBS, and kept in 750 µL isopropanol and left overnight. The tissues were then transferred to 96-well plate and OD values were obtained at 570 nm. The mean percent viability was then calculated.

#### 4.2.13. Stability Study

The modified ICH guidelines for stability studies were followed [36].The optimized formulations of COG were stored at different temperatures and humidity conditions and assessed for appearance, phase separation, pH, and transmittance percentage.

## Figures and Tables

**Table 1:** Thermodynamic stability studies of selected nanoemulsion formulations.

Oil:Smix	Heating–Cooling Cycles	Centrifugation	Freeze–Thaw	Inference
1:5	Clear	Clear	Clear	Stable
1:6	PS	PS	PS	Unstable
1:7	PS	PS	PS	Unstable
1:8	PS	PS	PS	Unstable

**Table 2:** FT-IR spectra peaks of chamomile oil and nanoemulsion. The figures represent the wave number values for different functional groups.

S. No	Groups	Actual Value (cm^−1^)	Observed Values	
			Chamomile Oil	Nanoemulsion
1.	-CH_2_- (aliphatic asymmetric)	2926	2929.21	2925.55
2.	C=O	1730–1750	1731.81	1732.42
3.	=C-H (Scissor)	Approx. 1465	1456.51	1457.14
4.	C-O	1000–1300	1374.96	1355.23

**Table 3:** Drug-release kinetic analysis of the nanogel formulation using different models.

Zero-Order		First-Order		Higuchi		Korsmeyer–Peppas			Hixson–Crowell	
K_0_ (intercept)	R^2^	K_1_ (intercept)	R^2^	K_H_ (intercept)	R^2^	K_KP_ (intercept)	N	R^2^	K_HC_	R^2^
0.054	0.1065	0.001	0.1124	0.237	0.8763	0.290	0.417	0.9051	0.000	0.1104

**Table 4:** The time for latency before treatment and post-treatment using different formulations during the tail flick test.

Groups	Dose	Time (S)		Percent Inhibition
		Pre-Treatment	Post-Treatment	
Control (saline)	1 mL/kg, IP	2.88 ± 0.75	2.81 ± 1.12	-
Standard treatment (diclofenac)	20 mg/kg, IP	2.77 ± 0.31	7.21 ± 0.97	36.3
Test treatment (COG)	100 mg/rat, topical	2.58 ± 0.93	6.88 ± 0.81	34.62

**Table 5:** The writhing count exhibited as a result of treatment with different formulations during the acetic acid-induced writhing test.

Groups	Dose	No. of Writhes	% Protection
Control (saline)	0.3%, 10 mL/kg Ip	24.88 ± 0.98	
Standard (diclofenac sodium)	20 mg/kg Ip	11.27 ± 1.1	54.7%
Test (COG)	100 mg/rat, topical	14.75 ± 0.95	40.71%

**Table 6:** Stability studies of optimized nanogel at different time intervals and temperature/humidity conditions.

Storage Conditions		Parameters			
Time	Temperature/Relative Humidity	Appearance	Phase Separation	pH	Percent Transmittance
0 day	25 ± 2 °C/60 ± 5% RH	Good	No	5.9	96.33%
	40 ± 2 °C/75 ± 5% RH	Good	No	5.9	96.41%
30 days	25 ± 2 °C/60 ± 5% RH	Good	No	5.8	95.18%
	40 ± 2 °C/75 ± 5% RH	Good	No	6.0	95.12%
60 days	25 ± 2 °C/60 ± 5% RH	Good	No	6.1	95.89%
	40 ± 2 °C/75 ± 5% RH	Good	No	6.0	95.93%

## Data Availability

The research data will be supplied on request.
